# Perceptions of the effects of armed conflict on maternal and reproductive health services and outcomes in Burundi and Northern Uganda: a qualitative study

**DOI:** 10.1186/s12914-015-0045-z

**Published:** 2015-04-03

**Authors:** Primus Che Chi, Patience Bulage, Henrik Urdal, Johanne Sundby

**Affiliations:** Peace Research Institute Oslo, PO Box 9229, Grønland, Oslo Norway; Institute of Health and Society, University of Oslo, PO Box 1130, Blindern, Oslo Norway; International Organization for Migration, Plot 6A, Naguru Crescent, Kampala Uganda

**Keywords:** Armed conflict, Effects, Access to healthcare, Quality of healthcare, Maternal health, Impact, Health system

## Abstract

**Background:**

Armed conflict potentially poses serious challenges to access and quality of maternal and reproductive health (MRH) services, resulting in increased maternal morbidity and mortality. The effects of armed conflict may vary from one setting to another, including the mechanisms/channels through which the conflict may lead to poor access to and quality of health services. This study aims to explore the effects of armed conflict on MRH in Burundi and Northern Uganda.

**Methods:**

This is a descriptive qualitative study that used in-depth interviews (IDIs) and focus group discussions (FGDs) with women, health providers and staff of NGOs for data collection. Issues discussed include the effects of armed conflict on access and quality of MRH services and outcomes, and the mechanisms through which armed conflict leads to poor access and quality of MRH services. A total of 63 IDIs and 8 FGDs were conducted involving 115 participants.

**Results:**

The main themes that emerged from the study were: armed conflict as a cause of limited access to and poor quality of MRH services; armed conflict as a cause of poor MRH outcomes; and armed conflict as a route to improved access to health care. The main mechanisms through which the conflict led to poor access and quality of MRH services varied across the sites: attacks on health facilities and looting of medical supplies in both sites; targeted killing of health personnel and favouritism in the provision of healthcare in Burundi; and abduction of health providers in Northern Uganda. The perceived effects of the conflict on MRH outcomes included: increased maternal and newborn morbidity and mortality; high prevalence of HIV/AIDS and SGBV; increased levels of prostitution, teenage pregnancy and clandestine abortion; and high fertility levels. Relocation to government recognised IDP camps was perceived to improve access to health services.

**Conclusions:**

The effects of armed conflict on MRH services and outcomes are substantial. The mechanisms through which armed conflict leads to poor access and quality of MRH services vary from one setting to another. All these issues need to be considered in the design and implementation of interventions to improve MRH in these settings.

**Electronic supplementary material:**

The online version of this article (doi:10.1186/s12914-015-0045-z) contains supplementary material, which is available to authorized users.

## Background

Armed conflict has been described as a public health problem due to its negative impact on health systems and population health [1]. One of the most affected components has been access to basic health services, including maternal and reproductive health (MRH) services. Bosmans et al. [2] found that the conflict in the Palestinian territories has resulted in decreased access to antenatal care (ANC) and postnatal care; increasing number of home deliveries, induced deliveries, deliveries at military checkpoints; and increased occurrence of gender-based violence. Still within these territories, Clark et al. [3] observed a very strong association between political violence and intimate partner violence. Kabakian-Khashodan [4] observed that during the 2006 war in Lebanon women reported interruptions in regular maternity care and experienced more pregnancy related complications. Relatedly, Price and Bohora [5] found a strong negative correlation between the incidence of conflict-related violence and the number of ANC consultations in Nepal. Armed conflict may equally increase vulnerability of women and girls by exposing them to increased risk of infection with HIV and other sexually transmitted infections, through sexual violence and absence of access to healthcare and testing [6]. Fatusic et al. [7] found that during the conflict in Bosnia and Herzegovina, the perinatal and maternal mortality increased, with most maternal deaths associated with an increased number of uterine ruptures, sepsis and bleeding due to shell injury of pregnant women. Other reported deteriorating effects of armed conflict on maternal and reproductive health include increased maternal mortality and total fertility [8], increased neonatal mortality [9], birth defects and pre-term births [10], and low birth weight [9,10] amongst others.

Burundi and Northern Uganda are both recovering from brutal civil wars that each claimed tens of thousands of lives^a^ and caused millions of people to be displaced [11,12]. The most recent Burundian civil war lasted from 1993 to 2005. The conflict had a strong ethnic character, involving the country’s two major ethnic groups, the Hutu and the Tutsi. On the other hand, the armed conflict in Northern Uganda started as an insurgency launched by the rebel Lord’s Resistance Army against the Ugandan national army. The war lasted from 1986 until 2006 and was described by Jan Egeland, the then UN Special Representative for Humanitarian Affairs, as ‘the worst forgotten humanitarian crisis in the world’ [13]. Although the conflict appears to have been rooted in mistrust and perceived marginalization of the Northern region by the government [14], it did not have a strong ethnic outlook.

Furthermore, the patterns of displacement caused by both conflicts were markedly different. During the more than two decades of conflict in Northern Uganda the population was mainly internally displaced. It is estimated that up to two million people were internally displaced and in those districts where most people were affected, up to 90% of the population had to be relocated to internally displaced persons (IDPs) camps [11]. In the case of Burundi, the conflict resulted in massive displacement of the population to neighbouring countries as refugees, especially in Tanzania and the Democratic Republic of Congo while a large number was also internally displaced within Burundi. By 1999, the number of Burundian refugees in Tanzania was estimated at 470, 000, representing more than 7% of the population of that country at the time [15]. Of the hundreds of thousands that were internally displaced, it is estimated that about 281, 000 were living in IDP camps by 2003 [16]. Reports suggest that the living conditions in the IDP camps in both Burundi and Northern Uganda were deplorable; characterized by overcrowding and insufficient access to food, water, sanitation and healthcare [11,16,17].

Nowadays, while specific and reliable maternal health data from Northern Uganda is difficult to obtain, the trends in national-level data from Uganda and Burundi for some MRH indicators provide a mixed picture when compared to the sub-Saharan Africa (SSA) average (Table 1). On the positive side, the proportion of births attended by skilled personnel and the antenatal care (ANC) coverage for at least one visit is above the average for the SSA region. However, ANC coverage for at least four visits and the prevalence of current contraceptive use among married women of reproductive age (especially for Burundi) remains lower than the regional average. Also the unmet need for family planning is poorer and the total fertility rate higher than the regional average. Additionally, the trends in the evolution of the indicators are overwhelmingly positive, except for the proportion of unmet need for family planning that has stagnated and remained relatively high in both Uganda and Burundi. In fact for Burundi, the proportion for unmet need for family planning has actually increased.

**Table 1:** **Trends of selected MRH indicators**

**Country/Indicators** ^**¤**^	**Burundi**	**Uganda**	**Sub-Saharan Africa**
Total population (thousands)^§^			
2000	9 233	24 276	638 974
2010	10 813	33 987	831 464
Total fertility rate*			
2000	7.1	6.9	5.8
2012	6.1	6.0	5.1
Maternal mortality ratio:			
2000	1000	650	830
2013	740	360	510
Proportion of births attended by skilled health personnel (%):			
2000/2001	25.2	39 (2001)	43
2010/2011	60.3	57.4 (2011)	45
Current contraceptive use among married women 15-49 years old (%):			
2000/2001	15.7	22.8	18.7
2011	21.9	30	25.1
Antenatal care coverage (at least one visit and at least four visits) (%):			
2000/2002	78, Unavailable	92.4; 41.9	72; 48
2010/2011	98.9; 33.4	93.3; 47.6	79; 49
Unmet need for family planning (%):			
2001/2001	29	35	26.5
2011	32.4	34.3	25.3
Infant mortality rate (0-1 year) per 1,000 live births:			
2000	91.6	89.1	94
2010	60.9	51.3	68

^¤^Figures are the most recent available data from the UN MDG indicators monitoring database, except otherwise stated: http://mdgs.un.org/unsd/mdg/Data.aspx.

^§^UN Population Division’s Population Estimates and Projection section: http://esa.un.org/unpd/wpp/unpp/panel_population.htm.

*Fertility rate, total (births per woman) - The World Bank: http://data.worldbank.org/indicator/SP.DYN.TFRT.IN.

Along with the growing advocacy to prioritize the reproductive health of women and girls in crisis settings [18,19] has come a closely related perennial problem: the unavailability of appropriate data and information to help inform policy development and implementation. Most studies on the impact of conflicts on maternal and reproductive health in such settings have largely been quantitative in design. While such studies are highly relevant in appreciating the trends of important health indicators such as the uptake of services, and prevalence and incidence of specific conditions, understanding the drivers of such trends or patterns may be more readily analysed through qualitative studies. This article therefore seeks to explore how armed conflict may lead to limited access to and quality of MRH services, from the perspectives of local health providers and staff of NGOs working in the domain of MRH, and women of reproductive age in Burundi and Northern Uganda. It constitutes part of a series of articles broadly exploring the state of maternal and reproductive health in conflict and post-conflict settings. The other articles focus on the determinants of women’s utilisation of MRH services [20] and the barriers to the delivery of quality emergency obstetric and neonatal care services. By comparing two armed conflicts with different patterns and lengths, and involving different stakeholders in the MRH landscape, we aim to provide a more comprehensive picture of the perceived effects of armed conflicts on MRH services and outcomes. Additionally, the study sites have experienced about the same period of time since the end of conflict. With our recent quantitative study [8] suggesting a negative impact of armed conflict on MRH outcomes (fertility and maternal mortality), this study adopts a qualitative approach to further explore the different mechanisms that lead to limited access to and poor quality of MRH services, and the effects this has on health outcomes. Our main research question is ‘*What are stakeholders’ perceptions of the effects of armed conflicts on maternal and reproductive health services and outcomes in Burundi and Northern Uganda?’*

## Methods

### Study settings

Data was collected from two provinces in Burundi, namely Bujumbura Marie and Ngozi and Gulu district in Northern Uganda. Participants in Burundi were recruited from the cities of Bujumbura and Ngozi and the communes of Ruhororo and Kinama, while in Gulu, participants were recruited from the sub-counties of Koro, Bobi and Bungatira and Gulu municipality (made up of four sub-counties: Pece, Layibi, Bar-dege, and Laroo).

### Study participants

Study participants were recruited from staff members of local and international non-governmental organizations (NGOs) and local health providers (nurses, midwives, doctors and senior administrators) working in the domain of MRH, and women of reproductive age, living in rural and semi-urban areas. Since we were interested in capturing the effect the conflict had on MRH outcomes and services, the NGOs and health providers invited to participate in the study had developed, supported and/or provided such services during the conflict, while the women had lived in the area during the crisis.

### Data collection method

This is a descriptive and explanatory qualitative study that used semi-structured in-depth interviews (IDIs) and focus group discussions (FGDs) for data collection. Interviews and FGDs were conducted in the local languages, French or English (where applicable) by the principal investigator (PCC) or trained local research assistants. Prior to the study, our target number of interviews and FGDs in each of the study sites was 10 IDIs and 1 FGD for each category of study participants. However, while on the field we observed that it will be logistically challenging to organise one FGD for our women category of participants, who live in different counties or communes. As such, we decided to organise two FGDs for these women in each of our study areas, with each FGD comprising of women living in the same county or commune. Data collection was therefore stopped when we had attained the target number of interviews and FGDs. A total of 63 IDIs and 8 FGDs were conducted. The fieldwork took place from June – September 2013.

### Issues discussed

The interviews and FGDs focused specifically on how the past armed conflict affected the general state of MRH, in the process exploring the negative consequences the conflict had on MRH services and the various channels through which the conflict led to limited access to and poor quality of health services. A sample of some of the questions posed to the respondents included: ‘*How did the war affect the accessibility to, affordability of and quality of MRH services?; Describe some eyewitness accounts of the negative consequences of the war on MRH; Can you describe your experience in accessing MRH services at your local health facility: (a)before, (b)during and (c)after the war?*’ The detailed guides for the interviews and FGDs for each of the participant categories have been published elsewhere [20].

### Data management and analysis

Interviews and FGDs with local health providers typically lasted from 50 – 130 minutes while those for the women lasted from 35 – 90 minutes. All interviews and FGDs were audio-recorded and later transcribed and translated into English (where applicable). Three team members open-coded the transcripts on QSR Nvivo (QSR International, 2012) and Microsoft® Word (where the texts of interest are highlight and the code first labeled using the ‘*New Commen*t’ sub-menu under the ‘*Review*’ menu). Microsoft® Word was used for coding and analysis by one of the co-authors who did not have access to Nvivo. The codes were descriptions or labels of specific ideas as the transcripts were read. Two team members reviewed the codes that were developed and the inter-coder reliability was high. Inter-related or similar codes were then clustered into different categories, and the categories were subsequently grouped into specific themes. We used the framework method [21], combining both the deductive and inductive approaches in the data. This allowed us to explore the main themes covered in the interviews and FGDs while being open to other unexpected aspects of participant experiences. There was therefore a constant interplay between data collection, analysis and theme development, where re-occurring unexpected themes were further explored in subsequent interviews and FGDs.

### Ethical considerations

Ethics and administrative approvals were obtained from the relevant authorities in Norway, Burundi, and Uganda. Ethics approval for the study was obtained from the following ethics committees: Regional Committee for Medical and Health Research Ethics, South-East (Norway), ‘le Comité National d’Ethique pour la Protection des êtres Humains Participant à la Recherche Biomédicale et Comportementale’ (Burundi), and Gulu University Institutional Review Committee (Uganda). All participants gave their informed consent before participating in the study and their anonymity, privacy and confidentiality was respected. Written or oral consent were appropriate and acceptable for our settings and approved by the relevant ethics committees.

The detailed and comprehensive methodology of the study, compliant with the COREQ and RATS reporting guidelines can be found at the additional file section below (Additional file 1).

## Results

One hundred and fifteen participants took part in the interviews and FGDs: 46 ‘women’, 32 ‘local health providers’ and 37 NGO staff. Among the ‘women’ participants their ages ranged from 17 to 55 years, with over 90% being in their reproductive age and all of them had given birth during the conflict and/or in the immediate post-conflict period. Their primary occupations were farming, ‘petit’ trading and housewife. The local health providers included community health workers, clinical officers, nurses, midwives and medical doctors (general practitioners and specialists) working in public and private health facilities and the local office of the ministry of health as administrators. The local health providers were categorised into: ‘LHP-Policy maker’ (senior administrative officials working at the local ministry of health) and ‘LHP’ (the rest of the local healthcare providers). The NGO staff members were recruited from 17 organisations across the study sites and included both medical and non-medical personnel. The organisations they worked for ranged from local/community-based, national to international in their operations and were involved in the delivery or support of MRH in the study areas. The NGO participants categorised into three sub-categories: NGO-Health provider (an NGO that also provides health services), NGO-Policy maker (UN-based NGO), and NGO (the rest of the NGOs). Of the 69 local health providers and NGO staff, 30 were males and 39 females. The distribution of interviews and FGDs with respect to the study sites and participant groups is shown on Table 2.

**Table 2:** **Number of interviews and FGDs, by study site and participant category**

**Country**	**Study areas**	**Participants**			**Total**
		**Local health providers**	**NGOs**	**Women**	
Burundi	Bujumbura Marie and Ngozi provinces	9 Interviews & 1 FGD	11 Interviews & 1 FGD	11 Interviews & 2 FGDs	31 Interviews & 4 FGDs
Uganda	Gulu district	12 Interviews & 1 FGD	10 Interviews & 1 FGD	10 Interviews & 2 FGD	32 Interviews & 4 FGDs
All countries		21 Interviews & 2 FGDs	21 Interviews and 2 FGDs	21 interviews & 4 FGDs	63 Interviews & 8 FGDs

The main themes that emerged from the study were: armed conflict as a cause of limited access to and poor quality of MRH services; armed conflict as a cause of poor MRH outcomes; and armed conflict as a route to improved access to health care. The themes will be explored in detail in the paragraphs that follow, and are supported by quotes from the interviews and FGDs from the different groups of study participants.

### Armed conflict as cause of limited access to and poor quality of maternal and reproductive health services

Participants across the sites strongly perceived that the armed conflict had a major negative effect on the health system in general, mainly through limited access to and poor quality of MRH services. The participants also highlighted the ways through which the conflict led to limited access to and poor quality of MRH services.

In all the study sites, especially in Burundi, the destruction of health facilities coupled with the looting of medical supplies and equipment, targeted killing and abduction of health providers, and eventual migration of health providers were well acknowledged channels through which the conflict lead to limited access to and poor quality of MRH services. With the health infrastructure and personnel under attack, access to the services they provided was not only disrupted or terminated, but the quality was also compromised.

“*The conflict affected this. If we start with the quality, there was first of all the lack of personnel, there were no medical supplies, the distance between functional health facilities increased because there were health structures that were closed or destroyed, and there was also lack of medicines and materials”.* LHP-Policy maker, IDI – Ngozi (LHP 17)

“*Many health personnel, including nurses and doctors were killed, while others fled to other countries. There were regions that were left without health personnel*”. LHP, FGD – Bujumbura (LHP 20)

While the targeted killing of health providers was often reported among participants in Burundi, in Northern Uganda reports of abduction of health providers by rebels was a more common occurrence.

“*Rebels wanted food, they needed sugar, they needed money, they needed medicine, drugs and they needed the health workers also; people to administer them the medicines. And they would get that from the hospital. So they would abduct the nurses”.* LHP, IDI – Gulu (LHP 5)

Furthermore, due to the very strong ethnic character of the Burundian conflict, the health providers that were left in the few health facilities which were operational during the conflict tended to provide services to clients based on ethnic origin as reported by many of the women. This was not however the case in Northern Uganda, where the conflict was not primarily of ethnic character. In addition, with medical supplies regularly looted at health facilities, the cost associated with seeking health services surged.

“*During the war, even health personnel did not treat persons in the same manner. They were sympathetic to persons of the same ethnic group. It was dangerous. Even services were very expensive because medicines were regularly looted* (from government health facilities)” Woman, FGD – Kinama (W 16)

**Figure 1:**
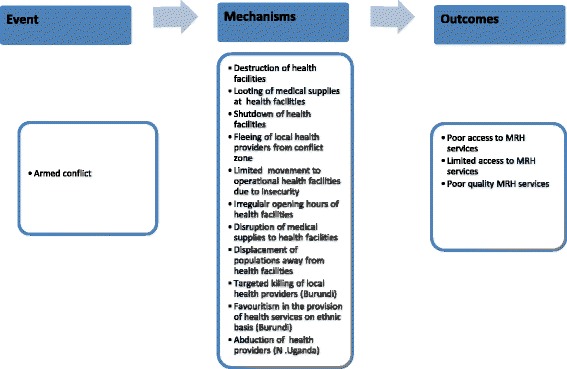
**Display of how armed conflict leads to limited access to and poor quality of maternal and reproductive health services.**

Summarily, while most of the channels were common across the study sites in Burundi and Northern Uganda, a few were specific to either site. The common mechanisms included the destruction of health facilities; the looting of medical supplies at health facilities; shutdown of health facilities; migration of local health providers away from the conflict zones; insecurity that prevented the movement of people to facilities that were operational; disruption of delivery of medical equipment and supplies; and irregular opening hours of health facilities. The mechanisms that were mainly common in Burundi were the targeted killing of health providers and favouritism in the provision of health services on ethnic basis, while the mechanism specific to Northern Uganda was the abduction of health providers by rebels. These mechanisms are illustrated in Figure 1 below.

Finally, the overall long-term impact of the conflict on MRH services was perceived to be the disruption infrastructural development and local training of health personnel, and poor retention of trained health personnel. For example, one of the respondents reported that while other regions of Uganda were investing resources in infrastructural and personnel development, the region of Northern region was set back due to the prevailing state of insecurity.

“*…the issue of challenges of not having enough facilities is quite attributable to the civil war which took place in this region. You will find that the twenty years or so when there was insecurity, there was a government policy that aimed at establishing health facilities as low as to the parish level and this could not happen because there was insecurity. There was no way you could go and build in a place where the rebels had camped. As such, some of these were halted. In some places they were training midwives. In other places they have even gone ahead to build better facilities through government and other donors but we missed out of these development initiatives* [in this region]”. NGO-Policy maker, IDI – Gulu (NGO 8)

Additionally, the general poor state of the health infrastructure and other social amenities has engendered the problem of poor retention of health personnel in the region of Northern Uganda.

### Armed conflict as a cause of poor maternal and reproductive health outcomes

The participants’ perceptions of armed conflict as a cause of poor MRH outcomes in the study areas was closely related to the negative immediate and long-term effects of the conflicts on access to and quality of MRH services discussed above. These perceived effects of the conflict on MRH outcomes included an increase in maternal and neonatal morbidity and mortality; high incidence of HIV/AIDS and sexual and gender-based violence; growing levels of prostitution, teenage pregnancy and clandestine abortion; and high fertility levels.

#### Increased maternal and newborn morbidity and mortality

One of the biggest casualties of MRH during the conflict in both study settings was the poor state of emergency obstetric care (EmOC). This service was largely unavailable for a huge segment of the population as health facilities in conflict-affected areas were closed and when opened they were poorly functional. Although these services were available in other parts of the country that were not engulfed by the conflict, the prevailing state of insecurity prevented pregnant women to travel and seek these services. The result was that maternal and neonatal morbidity and mortality reportedly increased.

“*During the war, it was dramatic because most of the women were in need of it* (EmOC)*. First of all, if for example one needed a caesarean section and she could not reach the health facility, she died or suffered from disability. I can say that nowadays if a caesarean section is available to about 7% of pregnant women, it went down to around 2-3% during the war. That meant about 4-5% of women in need of the service died or were disabled”.* LHP-Policy maker, IDI – Ngozi (LHP 18)

Additionally, access to facility deliveries and births attended by skilled personnel was disrupted during the conflict. The prevailing environment saw the re-emergence of traditional birth attendants (TBAs) as primary birth attendants in affected communities. Furthermore, pregnancy and delivery complications reportedly increased, especially as ANC attendance was largely a luxury for many women. Most of the participants felt that while those complications had been observed among pregnant women before, the incidence was higher during the conflict period.

One frequently reported maternal morbidity linked to the conflict in Burundi was an explosion in the cases of obstetric fistula during and immediately after the conflict due to inaccessibility to facility deliveries and skilled birth attendance for a large segment of the population. A number of participants described circumstances where many women with fistula found themselves abandoned and ostracized by their families or communities. Some husbands reportedly abandoned their wives because they had fistula.

Many women narrated incidents where they lost fellow pregnant women from pregnancy- and childbirth-related complications due to poor or no access to skilled services during the conflict. A number also recounted personal pregnancy and delivery challenges they went through at the height of the conflict that threatened their very survival.

“*When I gave birth to my first child, I had to run with blood on me to the bush. While giving birth to another child, rebels ambushed me, but one lady carried my child and ran to the other side and I almost died from bleeding, but thank God that they later on rushed with me home and saved my life with hot water*”. Woman, IDI – Gulu (W 3)

#### High prevalence of HIV/AIDS and SGBV

Across the study areas, most participants felt that the conflict brought about a surge in the prevalence of HIV and other sexually transmitted infections (STIs) as well as a high incidence of sexual and gender-based violence, including intimate partner violence. For example, the HIV/AIDS prevalence in Northern Uganda was reported at 11.9 – 12% compared to 7.3% for the national level. Similar concerns were reported by women in Burundi.

“*I think if it were not of the conflict, we would not have been affected by HIV/AIDS [*on this scale*]. Before the conflict we had not seen these things and we did not even know about them. However, after the conflict we have seen bad things such as incurable diseases, rape or sexual violence by a man who is older than you and who is like your parents or your grandfather. These things did not exist before the war. We can say that Burundians have lost their cultural norms*”. Woman, FGD – Kinama (W 18)

#### Prostitution, teenage pregnancy and clandestine abortion

Prostitution, especially among adolescents, was reported to be relatively widespread in all the study sites. Many respondents noted that most of the young women involved in the practice grew up in the displaced peoples’ camps and were exposed to sexual practices at a very early age. Also, some of them were orphans and/or heads of child-headed households who lost both or a single parent to the conflict.

“…*[T]he young children grew up in camps and were exposed to seeing bad and good things that have made them to get into sexual relationships very early and for that reason there are very many cases of child pregnancies. Apart from that there is prostitution; many of these young girls opt for sexual relationships just for money*”. LHP, IDI – Gulu (LHP 8)

Furthermore, some respondents reported that young girls who grow up without their parents and lacking other forms of support have been forced into survival sex and other risky sexual practices that have resulted in a growing problem of increasing teenage pregnancies. Midwives interviewed reported that teenage girls between 12-18 years from disadvantaged backgrounds are particularly vulnerable to unintended pregnancies and constituted over half of their clients.

With a high level of teenage and unwanted pregnancies, many women have resorted to clandestine and illegal abortion that in some cases ended in mortality or severe morbidity. Most health provider and NGO respondents were alarmed at what was considered growing phenomenon of abortion-related complications and deaths among young girls.

“*…from my experience the complications of abortion are there because there are many induced abortions which go unrecorded. There are many teenage pregnancies because these girls conceive without planning. They never intended and then later they do all sorts of manipulation to end the pregnancy and in so doing you find that they have perforated their uterus, they have contracted a lot of infections, they are bleeding and all that. These are very common things!*” NGO-Policy maker, IDI – Gulu (NGO 7)

#### High fertility rate

Most of the respondents, especially the NGO participants reported that the total fertility rate (TFR) increased strongly during the conflict. While a good number of women found it difficult to control their fertility due to lack of family planning services, others reportedly wanted to deliver more to replace their lost ones. This was particularly acute in Burundi where increasing the size of one’s own ethnic group was cited as an additional motivation.

“*It was an inter-ethnic conflict where one group stayed in while the other went out. …in both groups the goal was that of replacing the dead members…the idea was to have as many children as possible…*” NGO, IDI – Ngozi (NGO 15)

Although the conflict is over, the TFR across the study sites remains very high, a situation that most respondents associated with strong cultural and societal pressures on women to bear more children to replace the family members lost during the war. Culturally, a large family size is still considered a sign of wealth in some rural areas.

“*…you know people do not want to stop delivering. They say they have lost so many people because of the war and as such they want those women to deliver their aunts, their brothers and their uncles*…*Our people were killed during the war; we need to add more numbers*”. NGO-Health provider, IDI – Gulu (NGO 9)

Also, with the disruption of the health system during the conflict, the development of child health related services is still a considerable challenge with high levels of infant mortality. Some health provider and NGO respondents felt that this has also contributed to keeping the current fertility rate among the general population very high. Other reasons identified for the high fertility were the low use of modern contraceptives among the women due to poor support from their male-partners, mothers-in-law, and local religious and cultural groups, and the fear of severe side effects. In Burundi, some NGO and health provider respondents felt that the introduction of a selective healthcare policy that waives user fees for pregnant women and children below five has encouraged some men to ‘*just be making babies without thinking*’ as maternal and child care services are free of charge in public facilities.

### Armed conflict as a route to improved access to health care

While acknowledging the overall negative effects of the armed conflict on MRH in the study areas, a number of participants in Northern Uganda felt that the prevailing state of maternal health in the region was better for a segment of the population at the height of the conflict compared to the immediate post-conflict period. They attributed this to the influx of NGOs and international funding and resources into government-recognised IDP settlements to which some populations from remote villages were relocated. Health facilities were also constructed within some of those IDP settlements, facilitating access to basic health care for the populations within those camps.

“*…before the conflict health facilities were far away but when people gathered in camps the health facilities were concentrated within the vicinity of and access to the population. Long before, health facilities were far away from the population and the health workers were not easily accessible*.” Woman, IDI – Gulu (W 9)

“…*over the 20 year period, people have concentrated in places and if you notice the location of these health units you will see that they were camp settings. So health units were very easily accessible to them and health workers could move into the communities very easily…everybody was in the same place. Of course that posed other issues but at least for the physical access it ensured that the children were easily immunised and it was even easy to go door-to-door to find out whether a child has been immunized*”. NGO, IDI – Gulu (NGO 4)

However, the NGO respondents perceived that towards the end of the crisis and the beginning of recovery, general access to health care, including MRH services, was severely compromised as camp residents had to relocate to places with little or no access to health care and other basic amenities, resulting in an unanticipated new health crisis within the recovery.

“*…I know people (*Organizations*) at that time that left and little did they know that there would be a crisis in the recovery; we are here in this camp, where you are returning to, everything was destroyed. You go like 50 km away and what do you find? You find that the water sources were destroyed, the schools are not there, and there are no facilities. You are now leaving every social amenity or services where the camp is…Now it meant you had to open up roads, you had to put up structures and get the health workers as the health workers who were serving in those camps were basically from the NGOs, they were not from the government, a good number, almost 80%. ….So these are the critical gaps or the crises that we see in the recovery process*”. NGO-Policy maker, IDI – Gulu (NGO 7)

This phenomenon was not reported across our study sites in Burundi.

## Discussion

This study explores the perceived consequences of armed conflict on MRH services and outcomes in two post-conflict countries in sub-Saharan Africa. In the paragraphs that follow we discuss our main findings with respect to the existing literature on MRH services and outcomes in conflict, post-conflict, and other crisis settings.

### Armed conflict as the cause of limited access to and poor quality of maternal and reproductive health services

While armed conflicts are known to negatively affect access to and quality of health services, including MRH services, the main mechanisms vary from one setting to another. The main channels we observed in our study have also been reported elsewhere. In Nepal, Ghimire and Pun [22] have reported similar channels as a result of the Maoist insurgency, where health facilities were attacked and destroyed, rendering them inoperable, and some health providers were targeted by armed groups for perceived support of their adversaries. Furthermore, during the war in Bosnia-Herzegovina, health services, especially those supporting maternal and child health were badly disrupted, with about 35% of facilities destroyed or badly damaged [23]. In post-conflict Somaliland, Leather et al. [24] observed that the conflict also inflicted serious damage on the health infrastructure and caused the death and migration of many health workers. During the Chiapas armed conflict in Mexico, Brentlinger et al. [25] also reported a wide range of similar channels to those observed in our study. In addition, they found that ambulances were seldom available to transport patients to functional health facilities, and pregnant women who developed complications after sunset were rarely transported because travel at night was very unsafe, with harassment, assault, or extortion by armed persons at roadblocks a common phenomenon. Also, while we found reports of bias in the delivery of health services on ethnic basis at our study site in Burundi, the Colombian study also reported cases of bias, but on political and religious affiliation. Although reports of such practices are not widespread in the public health literature, healthcare delivery in some states emerging from ethnic conflict have also been shown to be segregated on ethnic lines [26-29]. Despite the fact that health providers are expected to be impartial in the provision of healthcare, it is challenging to establish effective mechanisms to enforce this during crisis situations like armed conflicts.

Furthermore, in the conflict in Eastern Myanmar, the targeting of medical staff, buildings, and supplies has also been reported [30]. The targeted killing of health care providers and attacks on health infrastructure during armed conflicts appears to be a growing and worrying tactic in modern day warfare. For example, a 2011 report [31] published by the International Committee of the Red Cross (ICRC) revealed that violent lethal attacks on patients, health care workers and facilities, and on medical vehicles are widespread in many conflict settings and pose a serious concern to accessing health care in such settings. Another ICRC report [32] showed that in 22 countries affected by armed conflicts and other emergencies, 921 violent incidents affecting health care were reported over the course of 2012, included 150 killings and 73 kidnappings of health care providers.

In our study, most of these mechanisms were common across the study sites but a few were unique to each study site. Notably, the channel of targeted killing of health providers and favouritism in the delivery of services was reported only in Burundi, while the mechanism of abduction of health providers was reported only in Northern Uganda. These differences appear to reflect aspects of the conflict and the level of coordination of health services within the affected areas. The very strong ethnic character of the conflict in Burundi may have engendered attack of health providers across ethnic lines, as health providers were perceived as key ‘lifesavers’. With such practices common, health providers who continued to provide services would have preferred to offer such services mainly in areas where they felt their safety and security was guaranteed, and that might be within their own ethnic community. In Northern Uganda, since the conflict was largely fought as an insurgency, with rebels constantly on the run, abducting health providers from nearby health facilities to cater to rebel health needs would appear to have been a war strategy.

With a strong likelihood that access to health facilities might be compromised during times of conflict, alternative strategies of delivering health care need to be explored by the relevant public health actors on the ground. These may include, but are not limited to, the provision of mobile health services to affected populations, and negotiation among the various warring parties for emergency ceasefires to enable health services to be provided to affected populations. Furthermore, the use of local community structures and organisations in the provision of basic health care could also be explored. This may include the training and equipping of traditional birth attendants and community health workers.

### Armed conflict as a cause of poor maternal and reproductive health outcomes

Findings similar to ours have been reported in other conflict and post-conflict settings. During the Chiapas armed conflict in Mexico, Brentlinger et al. [25] observed a substantial increase in the maternal and perinatal mortality ratios associated with increased home deliveries, and difficulty in accessing emergency obstetrical care. The authors also found that both home delivery and mortality were associated with higher levels of intra-community division based on political and religious affiliations, and the primary providers of ANC services were traditional birth attendants. In Eastern Myanmar where an armed conflict between various separatist groups and the military regime is ongoing, with very high levels of maternal mortality comparable to those in our study sites, Loyer et al. [30] associated this to a combination of health system-, conflict- and politically-related factors. They found that lack of access to and availability of maternal healthcare services is the main cause of maternal deaths. They also noted that while conflict-related factors are difficult to link directly to maternal deaths, these factors, especially human rights violations such as forced labour, soldier violence, theft or destruction of food supplies, injuries from landmines, and forced displacement appear to increase the risk of illness and death in pregnancy. Similar observations have been reported in other conflict and post-conflict regions including Sri Lanka [33,34], and Bosnia and Herzegovina [7]. O'Hare and Southall [35] have equally reported that sub-Saharan African countries that have recently experienced conflict have higher levels of maternal deaths, coupled with lower levels of skilled-attended births. These findings suggest that a key strategy to reduce maternal morbidity and mortality in such settings is to ensure access and availability of maternal health services, including quality emergency obstetric and neonatal care signal functions.

Our observations equally suggest that the protracted nature of the conflict in Northern Uganda has enhanced the spread of HIV/AIDS. The relationship between armed conflict and the transmission and spread of HIV remains complex and contentious. Iqbal and Zorn [36] found a positive relationship between armed conflict and the prevalence of HIV/AIDS among 43 African countries from 1997 – 2005. On the other hand, Spiegel et al. [37] have undertaken a systematic review on the prevalence of HIV infection in some conflict-affected and displaced people in seven countries in sub-Saharan Africa and their findings are contrary to those reported by Iqbal and Zorn. To better understanding the effect of conflict on HIV/AIDS prevalence Mock et al. [38] have proposed a number of contextual factors that may enhance the transmission and spread of the infection. These include increased interaction among military and civilians; increased levels of commercial or casual sex; decreased availability and utilisation of reproductive health and other health services; decreased use of means to prevent HIV transmission; and increased population mixing following large internal population movements among others. The presence of these factors in an area where the prevalence of HIV/AIDS is already high can serve to push up the prevalence in the general population. This seems to be the scenario that existed in Northern Uganda during the over two decades of conflict. A recent study undertaken in Northern Uganda supports our findings. Patel et al. [39] reported an overall prevalence of 12.8% among young men and women. According to Westerhaus et al. [40], the major factors that enhanced the spread of HIV/AIDS during the insurgency in Northern Uganda were the mass abduction of children into the rebel Lord’s Resistance Army (LRA), the phenomenon of night commuting, and the existence of IDP camps. Although concerns around HIV/AIDS were not very common among our participants in Burundi, one of the policy makers interviewed was concerned that this is a neglected domain that, if not well addressed, may turn into a serious problem in the future. With a number of contextual factors that could engender the spread of HIV and other STIs in both Northern Uganda and Burundi, including the current refugee crisis in South Sudan, effective interventions need to be put in place to stem the spread of these diseases.

The reported growing concern of SGBV observed in our study confirms earlier findings in Burundi and Uganda. A national survey on attitudes towards intimate partner violence in Uganda found that more than half of the men and about three-quarters of the women expressed attitudes that were supportive of wife beating [41]. The survey also found that half of married women had experienced intimate partner violence while 41% of men reported being perpetrators of intimate partner violence. A recent survey found that 27% of women in Kampala had experienced physical and sexual violence from an intimate partner [42]. The prevalence of SGBV in Burundi for the past years has also been high, a situation largely associated with the influx of returning refugees and displaced persons, the presence of large demobilized ex-combatants, a high prevalence of female-headed households, widespread lack of economic opportunities and a general breakdown in social norms [43]. Furthermore, the moral degradation and normalization of violence that characterised the war has led to a situation where many people perceive sexual violence as acceptable [44]. In another post-conflict setting in Liberia, Allen and Devit [45] have equally observed very high levels of intimate partner violence including physical abuse, sexual and verbal abuse and economic abuse, with some level of acceptability of these practices within the study population. While the main current perpetrators of SGBV in our study settings are intimate partners or someone within the family, during the conflict, violators also included warring actors. The reported high levels of SGBV in these sites might be linked to a growing knowledge gap between couples, where most educational interventions by NGOs and other policy makers have largely been focused on women and girls, leaving out the men in a disadvantaged or vulnerable position. A number of stakeholders expressed concerns that most interventions and programmes are disproportionately biased towards women. Another possible explanation for the high levels of SGBV might be that the economic empowerment programmes targeting women might have had the unintended effect of creating more assertive women who are the main providers for the household, creating a complex of inferiority within some men who might exercise this through SGBV. This is very much in agreement with a recent report on conflict analysis in Northern Uganda which observed that changing gender relations during and after the conflict have contributed to high levels of domestic and sexual and gender-based violence in the region [46]. While most traditional interventions to reduce SGBV have largely been focused on women’s empowerment through education/training, economic empowerment through micro-finance programmes, and the provision of legal support, it is also important to explore and address some of the root causes of this phenomenon on the side of the perpetrators. Providing interventions that can meaningfully improve the economic and psychological wellbeing of the perpetrators also needs some attention. Failure to explore and address these issues might only go to further entrench the existing pattern of abuse of women and the emergence of other forms of violence against women.

High levels of teenage pregnancy are a commonly reported problem in certain conflict and post-conflict settings. In some refugee camps hosting Burundian refugees in Tanzania, Märta [47] reported a similar pattern, with the high prevalence associated with low education attainment, breakdown of the culture, poverty, and unstable family relations. In post-conflict Liberia, Kennedy et al. [48] and Atwood et al. [49] observed that in-school adolescent girls were not only vulnerable to unplanned pregnancies, but also HIV/AIDS and other STIs; high levels of sexual activity, early sexual debut, and unprotected and risky sexual practices including transactional sex. Within some post-conflict areas in Eastern Uganda, Muhwezi et al. [50] have observed vulnerability to high-risk sexual behaviour among the population, including transactional sex, early and forced marriage, and sexual predation. They equally observed that these practices were associated with a high concentration of people in camps, where idleness and unemployment were very common. Rujumba and Kwiringira [51] and Westerhaus et al. [40] have equally linked the conflict in Northern Uganda to the growing phenomenon of prostitution reported in our study. They associated this with a rise in moral decadence, loss of property and livelihood, and increasing poverty that characterised life in overcrowded IDP camps. The economic hardship that characterised the war in Burundi has equally facilitated and perpetuated the practice of prostitution. With very high levels of violence against women and insecurity in Burundi during the war, some women, especially widows, perceived prostitution and concubinage as a safer option rather than being alone, where they may be more exposed and vulnerable to abuse and violence by unknown delinquents, armed gangs and bandits [52]. These findings strongly point to the long-term impact of conflict on the breakdown of social and cultural norms and practices, where practices that used to be considered culturally unacceptable are becoming the norm. Addressing such issues will require a complex set of interventions that not only address the root causes of such practices, but also the predisposing factors.

The high total fertility rate reported in our study was expected, as Burundi and Uganda currently have among the highest fertility rates in the world. A recent study [8] also found that sub-Saharan African countries that have recently experienced armed conflict have a higher fertility rate compared to those that have not experienced conflict. Our findings are also supported by a recent study in post-conflict Rwanda that reported an increase in total fertility following the genocide, and this was associated with a strong replacement effect among the general population [53]. A strong replacement effect, coupled with a strong cultural desire for large family size and a low uptake of family planning services could possibly account for the high total fertility within our study settings. The replacement effect may be stronger in ethnic conflicts, especially in contexts where groups are competing for population size as might arguably be the case in Burundi. Data from the Population Reference Bureau^b^, however, shows a slow and gradual decline in the total fertility rate in Burundi and Uganda from 1970 to 2013, with similar levels of decline across the period. From 1970 to 2013, TFR has only declined from 7.3 to 6.1 and from 7.1 to 5.9 in Burundi and Uganda respectively. This might suggest that the replacement effect is not only limited to ethnic conflicts.

### Armed conflict as a route to improved access to health care

On a positive note, we observed that a number of respondents from Northern Uganda felt that staying in a government-recognised IDP camp within the conflict-affected region enhanced their access to basic health services. This was because some of these camps were attached to a health facility and better resourced with support from humanitarian organisations. Some facilities even offered 24-hour services that also facilitated the management of emergencies. While this improved access to health care was limited only to a small segment of the population, especially those relocated from remote rural areas where access to basic healthcare was very poor before the conflict, a huge segment of the population experienced a deterioration in access to basic healthcare during the conflict. Chan and Kim [54] have also reported similar findings among smaller IDP camps in Kashmir, Pakistan following an earthquake. They observed that residents in smaller unofficial camps had worse health outcomes; had less access to information, medical services and medication; had the largest average family size; and received the least assistance and resources to sustain livelihood compared to residents in smaller official camps. However, among the larger IDP camps, the health outcomes were similar between the residents in the official and unofficial camps. This is the same phenomenon that some of our respondents in Northern Uganda experienced during the armed conflict, where residents in official IDPs camps had better access to health services than in their communities of origin prior to displacement. Relatedly, studies among refugee populations have also reported better health outcomes in camp populations compared to both non-camp populations in the area of refuge, and populations in the area of origin [55-57]. Additionally, Howard et al. [58] found that among Liberian and Sierra Leonean refugee camps in Guinea, the knowledge and use of contraceptives was much higher than for the populations at large in Liberia, Sierra Leone, or Guinea. These findings highlight the opportunities presented by official and government-recognised settlements for displaced populations as important platforms to facilitate access to health care for large populations, with relatively fewer logistic challenges. Furthermore, it also draws attention to the need to improve the delivery of health care to populations in non-camped and unofficial settlements.

Overall, our main findings and contributions to the research on MRH in post-conflict settings are as follows. Firstly, armed conflict is a major cause of limited access to and poor quality of MRH services and the mechanisms through which this plays out are context-specific and vary from one armed conflict to another. Consequently, initiatives to improve access to and quality of MRH services in conflict and post-conflict settings should consider the main contextual channels contributing towards this phenomenon. Secondly, armed conflict negatively affects a range of MRH services and outcomes at the health system and individual levels. In this regard, interventions or packages of interventions for improving MRH in conflict and post-conflict settings should be developed and implemented, taking these factors into consideration, focusing on the most badly affected services and outcomes. Thirdly, while contextual factors like living in a government-recognised and well-resourced IDP camp can engender the development of poor attitudes and practices at the individual and community levels, including poor and risky sexual practices, it can equally serve as a platform to substantially improve access to basic health services. As such, while ensuring the availability of health facilities within camp settings, it is important for camp planners to explore strategies that will reduce the mediating factors that engender the growth of poor attitudes and practices that may further endanger population health.

### Strengths and limitations of study

The effect of armed conflicts on MRH services and outcomes is an issue of global importance, especially as conflict-affected countries are lagging behind in the attainment of the MDGs. However, there is a relative lack of research on the issue. Working with study sites that experienced different forms of armed conflict provides a ‘richer’ and more extensive appreciation of the effects of armed conflict on MRH services and outcomes compared to limiting the study to one study site. Additionally, the choice of healthcare providers, policy makers, and staff of organisations involved in health systems support and strengthening as research participants, complemented by perspectives of MRH clients, provides a more comprehensive picture of how the conflict affected health outcomes and services. Each of these participant groups contributed their unique experiences to the study that could not have been achieved with only one category of participants.

Several limitations were identified. The women participants recruited for the study were living within the catchment areas of some local health centres or had regular weekly access to basic healthcare services through mobile outreach clinics. We were unable to recruit women participants in highly disadvantaged remote areas that were not regularly served with basic health services. As such the perspectives of that group of women are not well captured in our study. In addition, being a qualitative study with non-random sampling of research participants in sites with unique contextual factors, the findings are not necessarily generalisable to other conflict and post-conflict settings. Another limitation is the lack pre-conflict and conflict baseline data to enable us to better compare with the current situation. However, with the perennial problem of lack of reliable data in conflict-affected countries and a general poor state of data collection in our study sites, we could only depend of the perspectives of the research participants. Additionally, considering that the time between our interviews and FGDs and the end of the conflict is over 8 years, combined with the non-random sampling method to recruit the research participants, any assessment of the effects of conflict on MRH services and outcomes may be subject to recall bias.

## Conclusions

Our study has identified the perceived consequences of armed conflicts on MRH services and outcomes in Burundi and Northern Uganda. Armed conflict was blamed for limited access to and poor quality of MRH services, and the main mechanisms through which this happened varied across the study sites. The abduction of health workers by the rebel LRA was only reported in Northern Uganda while the targeted killing of health workers, and bias on ethnic basis in the delivery of healthcare services was only reported in Burundi. The overall effect was the disruption of infrastructural development and the training health personnel, combined with a poor retention rate of health personnel. The major perceived effects of the conflict on MRH outcomes include: increased maternal and newborn morbidity and mortality; high prevalence of HIV/AIDS and SGBV; high levels of prostitution, teenage pregnancy and clandestine abortion; and high levels of fertility. One positive story that emerged from the study was the perceived improvement in access to basic MRH services to some segments of population in Northern Uganda as a result of relocation from remote villages to government-recognised/official IDP camps which were better resourced and equipped with health facilities. In order to improve the effectiveness of interventions to improve MRH and women’s health in general in conflict and post-conflict settings, these issues need to be considered.

## Endnotes

^a^The Uppsala Conflict Data Program (UCDP) Conflict Encyclopedia: http://www.pcr.uu.se/research/ucdp/database/. According to the UCDP about 20,000 people were killed in the conflict between the Lord’s Resistance Army (LRA) and the government, while another 7-8,000 civilians were killed by the LRA. Similarly, in Burundi, 15-20,000 were killed in the fighting between the armed groups and the government, while another 12,000 were killed in ‘one-sided’ violence (against unarmed civilians).^b^www.prb.org.

## Additional file

Additional file 1: This is a detailed COREQ- and RATS-compliant description of the materials and methods used for undertaking the study. It includes a description of the study settings and participants, data collection, management and analysis methods, methodological orientation, collaborative partnership, research assistants and guides, recruitment of participants and ethical considerations.

## Abbreviations

MRH: Maternal and reproductive health
IDP: Internally displaced person
SGBV: Sexual and gender-based violence
IDI: In-depth interview
FGD: Focus group discussion
TFR: Total fertility rate
