# Supplementary material for: Perceptions of the effects of armed conflict on maternal and reproductive health services and outcomes in Burundi and Northern Uganda: a qualitative study
Source: BMC Int Health Hum Rights. 2015 Apr 3;15:7. doi: 10.1186/s12914-015-0045-z (PMC4392810; doi:10.1186/s12914-015-0045-z)
Supplement: Additional file 1: — This is a detailed COREQ- and RATS-compliant description of the materials and methods used for undertaking the study. It includes a description of the study settings and participants, data collection, management and analysis methods, methodological orientation, collaborative partnership, research assistants and guides, recruitment of participants and ethical considerations. [file 12914_2015_45_MOESM1_ESM.docx]

**Additional file 1**

**Methods**

**Introduction**

This paper is part of a two-country qualitative fieldwork looking at the impact of armed conflict on maternal and reproductive health (MRH) in some post-conflict settings in sub-Saharan Africa.

**Study settings**

Data was collected from two provinces in Burundi, namely Bujumbura Marie and Ngozi and a district in Northern Uganda (Gulu). Participants in Burundi were recruited from the cities of Bujumbura and Ngozi and the rural and semi-urban communes of Ruhororo and Kinama respectively, while in Gulu, participants were recruited from the rural sub-counties of Koro, Bobi and Bungatira and Gulu municipality (made up of four sub-counties: Pece, Layibi, Bar-dege, and Laroo).

**Study Participants**

Study participants were recruited from among non-governmental organizations (NGOs), local health providers and women. Our choice of our research participants was informed by their active involvement in the demand, delivery, support, design or organisation of MRH services during and after the conflict. The NGOs included local, national and international organizations working in the domain of MRH, be it at the level of policy support/ technical assistance, health system support and strengthening, delivery of health services etc. The international NGOs included UN- and the non-UN-based organizations. Participants within the NGO domain were mostly maternal and/ or reproductive health programme or project advisers, managers or coordinators in their organizations, and included medical and non-medical personnel. The local health providers were drawn from clinics, health centres and hospitals and included nurses, midwives and doctors working on maternal and reproductive health issues in their institutions, mainly in the maternity, antenatal care, and obstetric and gynecological units. Others also included senior administrators at ministries of health at the provincial, regional or district levels. The women were mainly in their reproductive age and settled in rural or semi-urban areas. Their primary occupation was farming and they lived within 5km from the nearest health centre or were served with basic health services once a week by a mobile health team from the main city. Since we were interested in also capturing the effect the conflict had on maternal health services, the NGOs and health providers invited to participate in the study had developed, supported and/or provided MRH services during the conflict or shortly after the conflict, while the women had sought or attempted to seek for such services as well during such periods. The research participants were unknown to the research team as no prior relationship had been established with potential participants before the study was undertaken.

**Data collection method**

This is a qualitative study that used semi-structured face-t-face in-depth interviews and focus group discussions (FGDs) for data collection. Interviews and FGDs were conducted in the local languages, French or English (where applicable) by the principal investigator (PCC) or trained local research assistants (RAs). A total of 63 interviews and 8 FGDs, involving 115 participants were conducted. The fieldwork took place from June – September 2013.

To enhance data saturation, our research participants were purposively selected to involve the main stakeholders in the demand, delivery and organization of MRH in the study settings. Within each participant group we made efforts to recruit participants who might have diverse experiences. For example, within the local health providers we recruited nurses, midwives, medical doctors working in different departments of the hospital or health centre as well as those who served as administrators in the local ministry of health. This strategy ensured that a wide spectrum of perspectives was captured during the interviews and FGDs.

**Methodological orientation**

Since we are interested to capture the consequences armed conflict has had on MRH, including the mechanism through which it leads to limited access to and poor quality of health services, we adopted a descriptive and explanatory methodological orientation in the data collection. The goal of qualitative descriptive studies is to provide a ‘comprehensive summary of events in the everyday terms of those events’ and in this regards, researchers conducting qualitative descriptive studies are expected to ‘stay close to their data and to the surface of words and events’ (Sandelowski 2000, p334)^[[1]](#footnote-1)^. It is recommended as a ‘method of choice when straight descriptions of phenomena are desired (Sandelowski 2000, p334). The design is particularly useful for capturing ‘straight and largely unadorned answers to questions of special relevance to practitioners and policy makers’ (p337).

**Issues discussed**

The interviews and FGDs focused specifically on how the past armed conflict affected the general state of MRH, in the process exploring the perceived negative consequences the conflict on the health and welfare of women and girls. We equally explored how the armed conflict led to poor access to and quality of health services. A sample of some of the questions posed to the respondents included: ‘*How did the war affect the accessibility to, affordability of and quality of MRH services?; Describe some eyewitness accounts of the negative consequences of the war on MRH; Can you describe your experience in accessing MRH services at your local health facility: (a)before, (b)during and (c)after the war?*’ The detailed guides for the interviews and FGDs for each of the participant categories can be found in the additional file section (additional file 1).

**Collaborative partnership**

Prior to arrival in the study area, we undertook an internet search of potential NGOs working in the domain of MRH in the area. We also approached prominent organizations/institutions working in the domain of MSRH in the area for a list of existing organizations/ institutions working on the ground in that domain. In Burundi, our local partners were UNFPA-Burundi and the Burundi University Research Centre for Economic and Social Development (CURDES) while in Uganda we worked closely with Reproductive Health Uganda (RHU). These partner organizations are involved in health systems strengthening and support in the various settings and will serve as veritable platforms for dissemination of study findings. In each of the settings, RAs were locally recruited.

**Recruitment**

Purposive sampling was the main sampling method employed. We were interested in potential participants deemed information-rich for the goal of the study. For the women, we mainly approached those who had had some experience of the conflict or the period immediately after the conflict. We were particularly interested in those who had given birth during any of those periods in order to capture the challenges they experienced in order to seek health care. For the NGOs and local health personnel, we specifically targeted those working in the domain of MRH and whose interventions/activities are designed with conflict or post-conflict challenges in mind.

Upon arrival at a local health institution (hospital, health centre or clinic) and NGO office with a letter of support from the local administrative and/or health office, permission was further obtained from the institutional head to approach an appropriate staff to participate in the study. Most often, the institutional heads chose the most appropriate staff to approach based on the criteria we provided. At health institutions, our participants were mainly drawn from the maternity, antenatal care, and obstetric and gynecological units while at NGOs our participants were mainly maternal and/or reproductive health programme coordinators or advisors.

Recruitment of women participants was mainly within communities in rural and semi-urban areas. Upon arrival in each community, the research team presented themselves to the local council office or community head, usually with a letter(s) of support from the district or provincial health office and/or local collaborating partner. The council or community then approved our entrance into the community, where we could approach local households and invite local women to participant in our study. Participants were informed about the study goal and procedure in the local language and were requested to ask questions for clarity. Only those that provided their consent following the information session participated in the study. At any point in time, we made an effort to clarify that we were independent researchers and not an NGO and as such were not involved in any way to provision of MRH services in the area.

About 10% turned down our invitation to participate in the study. The main reasons for the refusal among the LHPs and NGO staff were the absence of their supervisor to authorise participation, opposition to the use of an audio-recorder and high workload. In the case of the women, the main reason was lack of time/ engagement with household activities.

**Conducting interviews and FGDs**

All individual interviews and FGDs with women were held within the community, mainly in their homes or nearby in some community space. Interviews and FGDs for NGO staff and local health personnel were held mainly at their places of work, and lawn of a local hotel. All interviews in French and the local languages were undertaken by the trained local RAs while all the English interviews were undertaken by the principal investigator (PCC). The PI is a male and a PhD student in International Health, with a background in quantitative and qualitative research methods.

During the interviews and FGDs field notes were taken. These mainly captured observations such as the physical setting, human and social environment, informal interactions and unplanned activities, nonverbal communication, formal interactions, and other information of interest. The field notes were used to better appreciate the context in which the interviews and FGDs were undertaken. Soft drinks, tea or coffee was provided to FGD participants during the discussion. We also provided transport reimbursement to FGD participants. To maintain privacy and confidentiality, non-participants were not present during the individual interviews and FGDs.

**Research Assistants and guides**

In each of the settings, RAs and guides were recruited locally. A total of 6 RAs and guides were recruited and trained across the study sites. All RAs were trained in conducting interviews and facilitating FGDs, which include a couple of pilot interviews with potential participants. They were equally trained in transcription and translation of interviews and FGDs. Most of the RAs had past experience in undertaking such activities and had some basic knowledge of the research topic. The training included pilot interviews and transcription and translation of interviews. Most of the RAs had past experience in conducting interviews and facilitating FGDs. All RAs understood the local language(s) plus English and/or French and were educated up to the university level. In Burundi interviews and FGDs were mainly held in the French or Kirundi language, while in Northern Uganda they were held in the English or Luo language. Of the 6 RAs, three were males and three females.

**Data Management and Analysis**

Interviews and FGDs with local health providers typically lasted from 50 – 130 minutes while that for the women lasted from 35 – 90 minutes. All interviews and FGDs were audio-recorded and later transcribed and translated into English (where applicable). The transcripts were not returned to the participants. Three team members open-coded the transcripts on QSR Nvivo (QSR International, 2012) and Microsoft® Word (where the texts of interest are highlight and the code first labeled using the ‘*New Commen*t’ sub-menu under the ‘*Review*’ menu). Microsoft® Word was used for coding and analysis by one of the co-authors who did not have access to Nvivo. The codes were descriptions or labels of specific ideas as the transcripts were read. Two team members reviewed the codes that were developed and the inter-coder reliability was high. Inter-related or similar codes were then clustered into different categories, and the categories were subsequently grouped into specific themes. The themes were inductively and deductively developed: identified in advance and derived from the data. There was therefore a constant interplay between data collection, analysis and theme development, with new and dominant ideas that emerged in earlier interviews and FGDs being explored deeper in subsequent and later interviews and discussions. The theme development was jointly undertaken by three team members.

Data analysis was guided by the framework method, a method that ‘sits within the broad family of analysis methods often terms thematic analysis or qualitative content analysis’ (Gale et al., 2013, p2)^[[2]](#footnote-2)^. A peculiar feature of this analytical technique is that it identifies 'commonalities and differences in qualitative data, before focusing on relationships between different parts of the data, thereby seeking to draw descriptive and/or explanatory conclusions clustered around themes' (p2). In a qualitative study undertaken by a multidisciplinary research team and where the data is primarily made up of transcripts of semi-structured interviews, Gale et al. (2013) recommend this analytical technique as it allows the data to be interpreted in a multidisciplinary fashion.

**Ethical considerations**

Ethics approval for the study was obtained from the Regional Committee for Medical and Health Research Ethics, South-East (Norway); le Comité National d’Ethique pour la Protection des êtres Humains Participant à la Recherche Biomédicale et Comportementale (Burundi); and Gulu University Institutional Review Committee (Uganda). We also received permission from local administrative and health authorities. All participants/informants gave their informed consent before participating in the study and their anonymity, privacy and confidentiality was respected. Written or oral consent were appropriate and acceptable for our settings and approved by the relevant ethics committees.

1. Sandelowski M: **Focus on Research Methods Whatever Happened to Qualitative Description?** *Res Nurs Health* 2000, 23, 334-340 [↑](#footnote-ref-1)
2. Gale NK, Heath G, Cameron E, Rashid S, Redwood S: **Using the framework method for the analysis of qualitative data in multi-disciplinary health research**. *BMC Med Res Methodol* 2013, 13:117 [↑](#footnote-ref-2)
